# Two Lanthanide Metal–Organic Frameworks Based on Semi-Rigid T-Shaped Tricarboxylate Ligand: Syntheses, Structures, and Properties

**DOI:** 10.3390/polym11050868

**Published:** 2019-05-13

**Authors:** Yun-Shan Xue, Zhuo-Lin Chen, Youzhen Dong, Wei-Wei Cheng

**Affiliations:** 1 School of Chemistry and Environmental Engineering, Yancheng Teachers University, Yancheng 224051, Jiangsu, China; czling1998@163.com (Z.-L.C.); dongyouzhen@163.com (Y.D.); 2 State Key Laboratory of Materials-oriented Chemical Engineering, Nanjing Tech University, Nanjing 210009, China; 3 School of Chemistry and Bioengineering, Nanjing Normal University Taizhou College, Taizhou 225300, China; 20111006@nnutc.edu.cn

**Keywords:** lanthanide metal–organic frameworks, crystal structures, luminescent property, catalytic performance

## Abstract

By using a semi-rigid tripodal ligand 5-(4-carboxybenzyloxy)isophthalic acid (H_3_L) and lanthanide metal ions (Nd^3+^, Tb^3+^), two novel lanthanide metal–organic frameworks, namely, {[Nd_2_L_2_(DMF)4] DMF}_n_ (**1**), and {TbL(DMF)(H_2_O)}_n_ (**2**), were synthesized under mild solvothermal conditions and structurally characterized by X-ray single crystal diffraction. Compounds **1** and **2** are isostructural, in which L^3–^ ligands linked dinuclear lanthanide metal–carboxylate units to form non-interpenetrated 3D network with (3,6)-connected topology. Luminescent investigations reveal that compound **1** displays the near-infrared emission at room temperature, and compound **2** can be employed as selective probe for Cr_2_O_7_^2^^−^ anion in aqueous solution based on luminescence quenching. Moreover, compound **2** exhibits catalytic activity for cyclo-addition of CO_2_ and epoxides under relatively mild conditions.

## 1. Introduction

Metal–organic frameworks (MOFs) as a class of new crystalline materials have drawn considerable interest recently, because of their widely potential applications in the fields of heterogeneous catalysis [1,2,3,4], chemical sensing [5,6,7], gas separation and storage [8,9,10,11], magnetism [12], photocatalytic materials [13], etc. Compared to traditional zeolite materials, MOFs possess tunable surfaces and pores, which are adjustable by choice of metal centers and modification of organic ligands. Hence, the structural features of the organic ligands, e.g., flexibility, functionality, symmetry, could greatly affect the performance and structure of the MOFs. Among various organic ligands, semi-rigid polycarboxylate ligands have been demonstrated to be good candidates for the construction of multifarious MOFs, which could adopt versatile conformations to meet different coordination environmental requirements, leading to unpredictable and intriguing framework topologies. So far, a few functional porous MOFs based on semi-rigid polycarboxylates have been widely reported. Taking the above into account, we chose a semi-rigid tricarboxylate ligand 5-(4-carboxybenzyloxy) isophthalic acid (H_3_L). The ligand exhibits a great variety of coordination modes to metal ions, due to the rigid benzoic acid and isophthalic acid moieties. Moreover, the H_3_L features certain flexibility, owing to the presence of –CH_2_O– group that can twist around to meet the requirement of the coordination environment. Among enormous MOFs, lanthanide metal–organic frameworks (Ln–MOFs) have attracted increasing attention as special branch of functional MOFs because of their unique physical properties such as color-pure luminescence, long excited-state luminescence lifetimes, distinct stokes shifts, typical sharp emission peaks, and large paramagnetism [14,15,16]. The lanthanide ions are different from other metal ions, mainly included the following aspects: (i) lanthanide ions could emit light over narrow wavelength ranges and show high fluorescence quantum yields; (ii) the lanthanide family of ions usually has high coordination number, e.g., eight-, nine-, ten-, eleven-, and twelve-coordinated with oxygen to yield versatile clusters; and (iii) the luminescence of Ln^3+^ ions could be effectively sensitized when coordinated with suitable conjugated chromophores by tuning f–f or f–d electronic transition, the so-called “antenna effect” [17,18,19]. 

Chemical fixation and conversion of carbon dioxide (CO_2_) have been a hot area of research because it was promising on solving the issue of greenhouse effect primarily caused by rapidly increasing CO_2_ levels in the atmosphere. Great effort has been devoted to reusing CO_2_ to synthesize valuable raw materials, because CO_2_ is nontoxic, inexpensive, abundant, and important C1 building block. Some examples show that MOFs could be ideal catalysts in the cyclo-addition reaction of CO_2_ with epoxides to form cyclic carbonates, owing to abundant catalytic active sites formed by inorganic metal units and organic ligands (e.g., metal-centered Lewis acid sites, Brønsted sites), which play a significant role in catalysis reactions. Furthermore, MOF-based catalysts exhibit excellent separability and circulation, thus there would be a very potential heterogeneous catalyst and have good application prospects in industry [20].

With the above considerations in mind, two Ln-MOFs, {[Nd_2_L_2_(DMF)_4_] DMF}_n_ (**1**), and {TbL(DMF)(H_2_O)}_n_ (**2**), based on the semi-rigid tripodal ligand H_3_L were obtained (H_3_L = 5-(4-carboxybenzyloxy)isophthalic acid). Compounds **1** and **2** are isostructural, and have been determined by single-crystal X-ray diffraction. Luminescence explorations reveal that Tb^3+^-based emission peaks of compound **2** could be selectively quenched by chromium (VI) anion, which suggested that compound **2** could serve as a promising luminescent probe for chromium (VI) anions. In addition, the studies have shown that compound **2** could catalyze the cyclo-addition of CO_2_ and epoxides under mild conditions to form cyclic carbonates efficiently.

## 2. Experimental Section

### 2.1. Synthesis of Compound 1

A mixture of Nd(NO_3_)_3_·6H_2_ (0.035 g, 0.09 mmol) and H_3_L (0.020 g, 0.06 mmol) in DMF (2 mL), EtOH (0.5 mL) and 150 μL of HNO_3_ (2 mol/L) was sealed in a 25 mL Teflon-lined stainless steel autoclave. The mixture was heated at 80 °C for 48 h, and then cooled to room temperature. Purple block single crystals of **1** were collected in ca. 28% yield based on H_3_L. Elemental analysis for C_47_H_50_N_5_Nd_2_O_19_, calcd (%): C, 44.19; H, 3.95; and N, 5.48. Found (%): C, 43.28; H, 4.33; and N, 5.19.

### 2.2. Synthesis of Compound 2

A mixture of Tb(NO_3_)_3_·6H_2_O (0.035 g, 0.08 mmol), and H_3_L (0.020 g, 0.06 mmol) in DMF (2 mL) plus of H_2_O (1 mL) was sealed in a 25 mL Teflon-lined stainless steel autoclave. The steel autoclave was heated at 80 °C for 48 h, and then cooled to room temperature. Colorless block single crystals of **2** were collected in ca. 24% yield based on H_3_L. Elemental analysis for C_19_H_18_NO_9_Tb, calcd (%): C, 40.51; H, 3.22; and N, 2.49. Found (%): C, 39.87; H, 3.47; and N, 2.73.

## 3. Results and Discussion

### 3.1. Synthesis

Two title compounds **1** and **2** were synthesized by using solvothermal method, inspired by the paper by Ma et al. [21]. The semi-rigid ligand H_3_L and lanthanide nitrate salts are dissolved in a mixture of DMF, EtOH, and H_2_O. Then, the resulting mixture was left undisturbed at 80 °C for 2 days. The reaction system was then gradually cooled to room temperature. The difference between our work and the procedures followed by Ma et al. is that we have used lanthanide nitrate salts and Ma et al. have reported Ln-MOFs by using lanthanide chlorides. It is well-known that many factors can affect the formation of the final products during a specific solvothermal approach, such as the type of metal salts, counter-anions, reaction temperature, pH values, the type of solvent, and starting concentrations of reactants [22]. In our case, a series of contrast experiments have been performed to explore the influence of reaction conditions on identity and crystallinity of the final products. The results show that the type of metal salts play vital role in reaction system. We employed lanthanide chloride compounds to synthesize Ln-MOFs under different reaction conditions of Ma et al. However, the resulting crystals were already known complexes. In addition, the result of contrast experiments shows that a slight difference in temperature and the proportion of mixed solvent of DMF, H_2_O, and EtOH have less influenced on crystallinity of crystals.

### 3.2. Crystal Structure Descriptions

**Figure 1:**
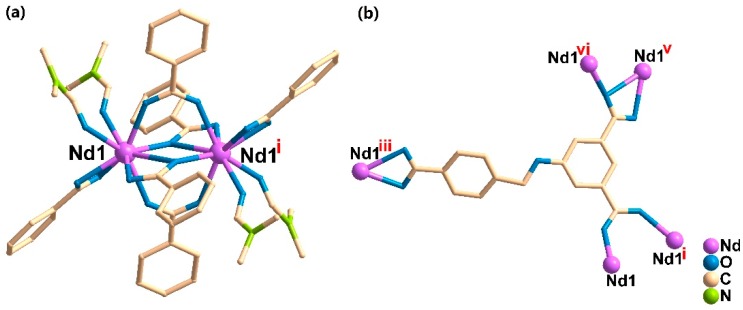
The coordination environment of (**a**) inorganic building units {Nd_2_(COOR)_6_(DMF)_4_} and (**b**) organic ligand L^3–^.

The single-crystal X-ray diffraction structure analyses indicate that compounds **1** and **2** are isostructural. Their differences lie in metal salts used (Nd and Tb for **1** and **2**, respectively). It should be noted that the coordinated solvent molecules in compound **2** are slightly different from compound **1**. As a representative example, only the structure description of compound **1** will be discussed here in detail. Compound **1** crystallizes in centrosymmetric monoclinic *C*2/*c* space group (No. 15, standard setting). Each asymmetric unit consists of one fully deprotonated ligand H_3_L, one crystallographically individual Nd(III) ion, two coordinated DMF molecules and one DMF solvent molecule (half-occupied). However, the asymmetric unit of compound **2** contains one L^3–^ organic ligand, one Tb(III) ion, one coordinated DMF molecule and one coordinated water molecule. As shown in Figure 1, the resulting three-dimensional (3D) framework of **1** is constructed from dinuclear metal–carboxylate cluster {Nd_2_(COOR)_6_(DMF)_4_} and organic ligand L^3–^ as building units. Each Nd center is nine-coordinated with two oxygen atoms from two bidentate carboxylate groups of two L^3–^ ligands, three oxygen atoms from two chelating bidentate carboxylate groups of the other two L^3–^ ligands, two oxygen atoms from one chelating carboxylate group of L^3–^ ligand (Nd–O distances ranging from 2.419(4) to 2.674(4) Å), and two oxygen atoms from two coordinated DMF molecules (Nd–O distance of 2.428(5) and 2.500(5) Å). The coordination environment of Nd center can be considered as a distorted monocapped square antiprism geometry (Figure 1a). Two neighboring Nd centers (Nd1 and symmetry-related Nd1A) are linked by two carboxyl groups in a bridging and chelating mode and two carboxyl groups via bidentate coordination mode to form dinuclear {Nd_2_(COOR)_6_(DMF)_4_} cluster, which connects six carboxylate groups from six independent L^3–^ ligands. Each fully deprotonated L^3–^ ligand adopts a *µ*_5_ coordination mode via three carboxyl groups linking metal clusters {Nd_2_(COOR)_6_(DMF)_4_} in bridging chelation, bidentate and chelation coordination modes (Figure 1b). In order to meet practical coordination configuration requirement, carboxyl groups rotate around the flexible alkyl bond and two benzene rings of L^3–^ are not in the same plane with dihedral angles of 17.48°.

**Figure 2:**
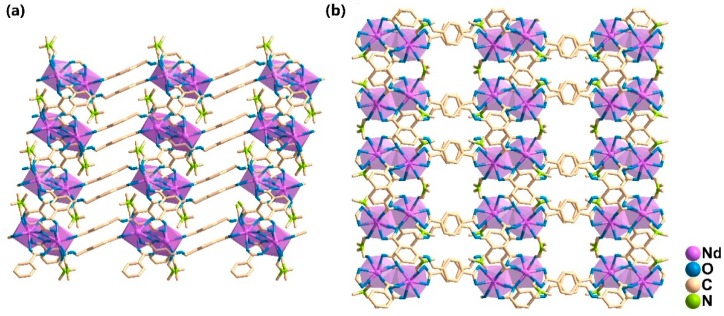
(**a**) The structure of **1** with channels along the ***b*** axe; and (**b**) the structure of **1** with channels along the ***c*** axe.

The rod-shaped clusters {Nd_2_(COOR)_6_(DMF)_4_} are interconnected by L^3–^ ligands to form a three-dimensional (3D) framework with two kinds of 1D channels along ***b*** (i.e., small and large rectangular channels) and ***c*** (i.e., small oval channels and large diamond channels) directions. As depicted in Figure 2, the coordinated DMF molecules protrude into the channels in the rectangular and diamond channels, resulting in reduced dimensionality of channels. These channels are occupied by guest DMF molecules, which could be located from the single-crystal X-ray diffraction data. The void volume is estimated by PLATON program [23] to be about 53.6% of the unit cell volume of 5566.9 Å^3^ (coordinated and guest DMF molecules not taken into account) and 22.6% of the unit cell volume (guest DMF molecule not taken into account).

**Figure 3:**
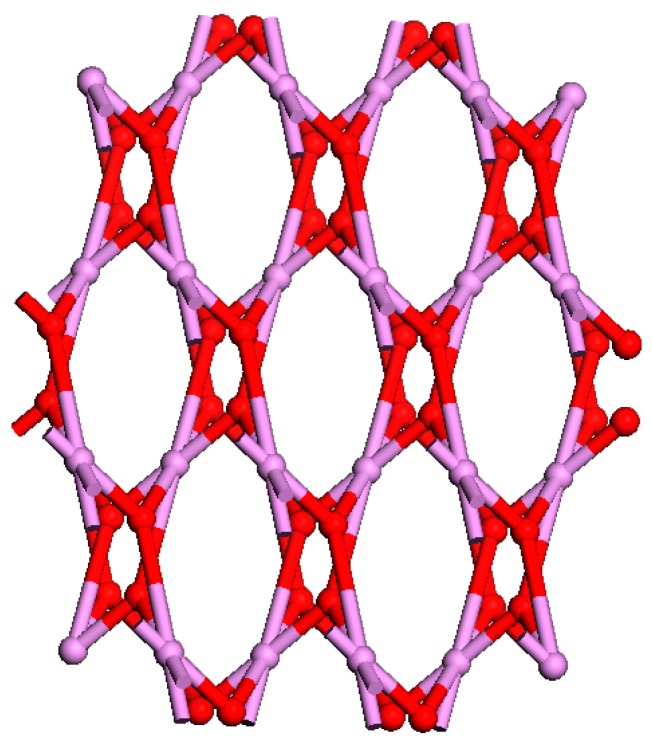
Schematic representation of the (3,6)-connected net of compound **1**.

To better understand the architecture of compound **1**, topological analysis (i.e., reducing multi-dimensional structures to simple node-and-linker nets) was performed using the TOPOS program [24]. It became evident that each L^3–^ ligand can be simplified as 3-connected node with point symbol {4^2^6}; the {Nd_2_(COOR)_6_(DMF)_4_} cluster is viewed as a pentagonal dodecahedron and considered as 6-connected node, each of which is connected by six L^3–^ ligands. On the basis of above simplification model, the whole framework of compound **1** could be simplified as 2-nodal (3,6)-connected net with the topology ***flu***-3,6-*C*2/*c*, which is a subnet of ***flu*** according to TOPOS analysis. The point symbol for net is {4^2^.6}_2_{4^4^.6^2^.8^7^.10^2^} (Figure 3).

### 3.3. Thermal Stability Analysis

Thermogravimetric analyses (TGA) were performed to investigate the thermal stabilities of compounds **1** and **2** using single crystal samples under N_2_ atmosphere. As shown in Figure S1, compounds **1** and **2** exhibited a similar profile. Compound **1** showed a weight loss of 27.6% at 20–450 °C, assigned to loss of guest and coordinated DMF molecules (calc. 28.5%). Further heating, the structure began to collapse and decomposed into metal oxide. TGA of compound **2** displayed a weight loss of 15.5% in the temperature range of 20–400 °C due to the removal of coordinated water and DMF molecules (calc. 16.3%). Higher temperature resulted in the collapse of the structure and decomposition of the organic ligand. 

### 3.4. Photoluminescent Properties

**Figure 4:**
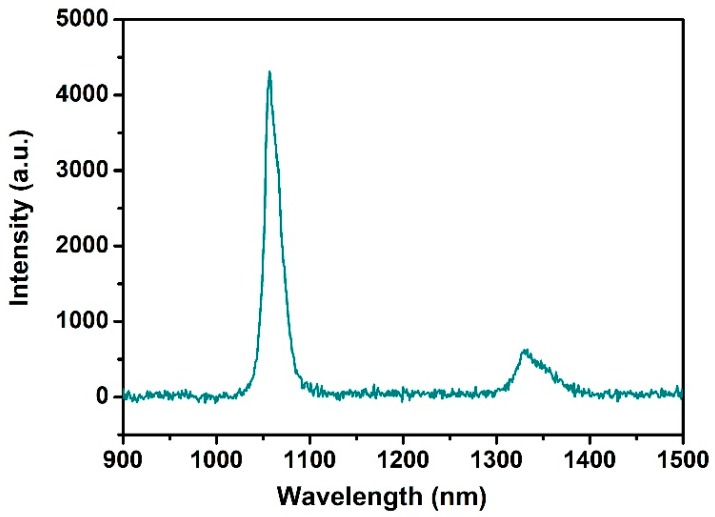
The NIR emission of compound **1** excited at 808 nm by laser.

The solid state photoluminescent spectra of compounds **1** and **2** were examined at room temperature. As illustrated in Figure S2, the free H_3_L ligand exhibits the maximum emission band at 363 nm (λ_ex_ = 290 nm), which is attributable to the π* → n or π* → π transitions [25,26]. The near-infrared (NIR) emission of the Nd^3+^ ion in compound **1** exhibits characteristic peaks at 1056 and 1330 nm upon excited at 808 nm by Nd:YAG laser, which can be assigned to the ^4^F_3/2_ → ^4^I_11/2_, ^4^F_3/2_ → ^4^I_13/2_ transitions of the Nd^3+^ ions, respectively [27,28] (Figure 4). 

Tb(III)-based compound **2** emits green luminescence visible to the naked eye under UV light (λ_ex_ = 310 nm) and exhibits the characteristic emission peaks for Tb(III) ion observed at 489, 544, 584, and 620 nm (Figure S3), which could be ascribed to the transitions of ^5^D_4_ → ^7^F_J_ (J = 6–3) [29,30,31]. The most intense emission peak corresponds to hypersensitive ^5^D_4_ → ^7^F_5_ transition at 544 nm [32,33]. The photoluminescent spectra of 2 exhibits characteristic emission bands of Tb(III) ion and the ligand-related emission peaks are not observable, which indicates energy transfer from H_3_L ligand to the Tb(III) ion during photoluminescence [34]. UV-Vis absorption spectra of compounds **1** and **2** are displayed in Figure S4. It is well established that the construction of Ln-MOFs not only have a significance to extend the Ln-MOFs family, but also allow exploiting them as fluorescent probes because of their remarkable luminescence properties and feature distinct advantages in sensitivity, operability and response time. Recently, Ln-MOFs employed as chemical sensors have been widely studied, and mainly focused on the fluorescence detection of small organic molecules [35,36], cations [37], and anions [38]. However, the detection of chromium(VI) anion species (Cr_2_O_7_^2^^−^ or CrO_4_^2−^) are poorly reported, which are commonly poisonous in wastewater and cause environment pollution and be harmful to the livings’ health. This thus urgently calls for more new luminescent sensors to detect chromium(VI) anions. 

**Figure 5:**
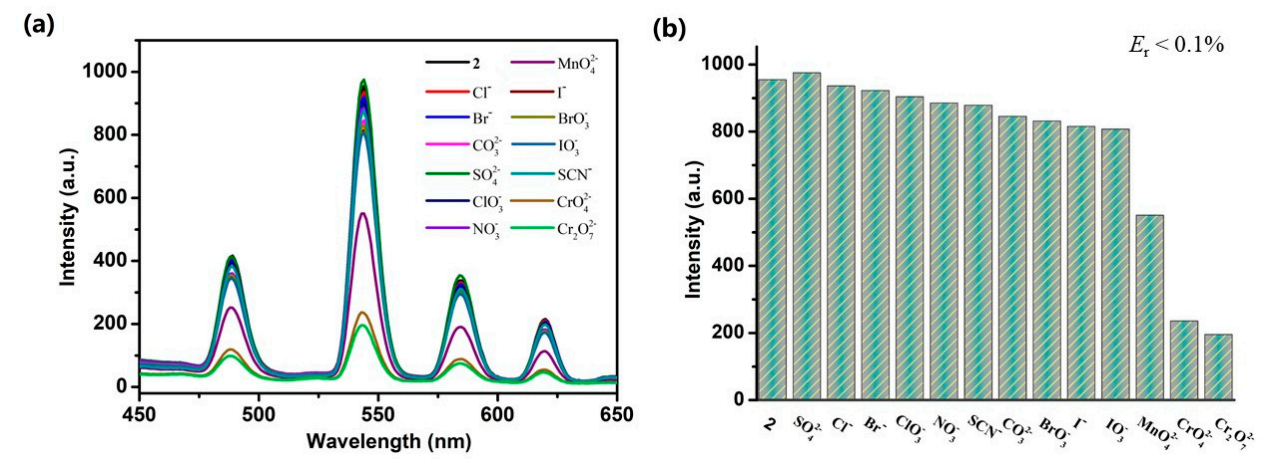
(**a**) The fluorescence spectra for compound **2** after the samples were dispersed in 0.005 mol/L concentration of aqueous solutions containing different metal ions; and (**b**) the enhancement or quenching efficiency histogram of compound **2**.

Based on the above, a series of spectroscopic experiments were conducted in water solutions including different anions to explore the sensing ability of compound **2**. The finely ground samples (10 mg) were dispersed in 1.9 mL aqueous solution by using ultrasonic method to form steady suspension solution, and then 100 μL K_m_X solution (0.1 mol/L, X = Br^−^, Cl^−^, I^−^, SCN^−^, IO_3_^−^, NO_3_^−^, BrO_3_^−^, ClO_3_^−^, SO_4_^2−^, CO_3_^2−^, MnO_4_^−^, Cr_2_O_7_^2−^, and CrO_4_^2−^) was slowly dropped into the above suspension solution (0.005 mol/L). These resultant suspensions were investigated by using fluorescence spectrophotometer. As illustrated in Figure 5a, the obtained fluorescence spectra still showed characteristic emission peaks of Tb(III) ion and only the strongest ^5^D_4_ → ^7^F_5_ transition of Tb^3+^ centered at 544 nm were recorded in Figure 5b. The results indicate that the emission intensities of different suspensions vary with anions. Obviously, most anions, such as halide ions, SCN^−^, IO_3_^−^, NO_3_^−^, SO_4_^2−^, CO_3_^2−^, ClO_3_^−^, and BrO_3_^−^ display no obvious or negligible influence on the intensity. The addition of MnO_4_^−^ lead to the fluorescence intensity slightly decrease, while Cr_2_O_7_^2−^, CrO_4_^2−^ exhibit significantly quenching behavior, indicating that compound **2** could act as a promising chemical sensor toward Cr(VI) in aqueous solution. Since the Cr_2_O_7_^2−^ anion showed the strongest quenching effect on the intensity of compound **2**, it was chosen as the representative to investigate the fluorescence sensing behaviors.

**Figure 6:**
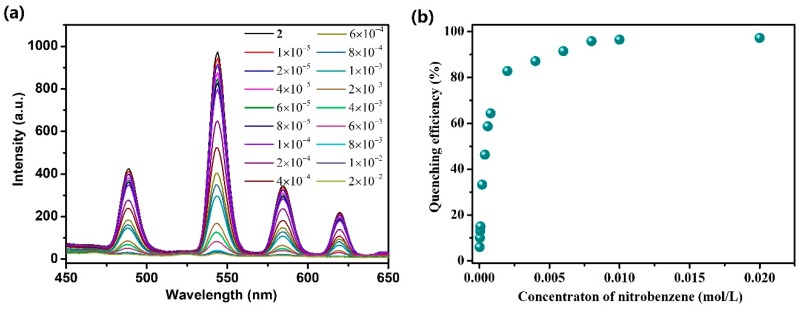
(**a**) The emission spectra of compound **2** dispersed in aqueous with different concentrations of Cr_2_O_7_^2−^; and (**b**) dependence of the quenching efficiency on the concentration of Cr_2_O_7_^2−^.

To explore the sensing sensitivity of compound **2** as Cr_2_O_7_^2−^ probe, different concentrations of Cr_2_O_7_^2−^ (10^–5^–10^–2^ mol/L) experiments were then performed in aqueous solution. The fluorescence intensity of compound **2** gradually reduced with increasing addition of Cr_2_O_7_^2−^, and the reduced intensity could be clearly distinguished with 1×10^−5^ mol/L Cr_2_O_7_^2−^ solution, indicating that detection limit of compound **2** could reach 10^−5^ mol/L as a fluorescent sensor for detecting Cr_2_O_7_^2−^ (Figure 6a). Moreover, the quenching efficiency was calculated by (*I*_0_ − *I*)/*I*_0_ × 100% equation, where *I*_0_ and *I* represent the intensity of compound **2** before and after adding Cr_2_O_7_^2−^, respectively. The quenching efficiency could reach 97.2% when the concentration of Cr_2_O_7_^2−^ increased to 0.02 mol/L (Figure 6b).

**Figure 7:**
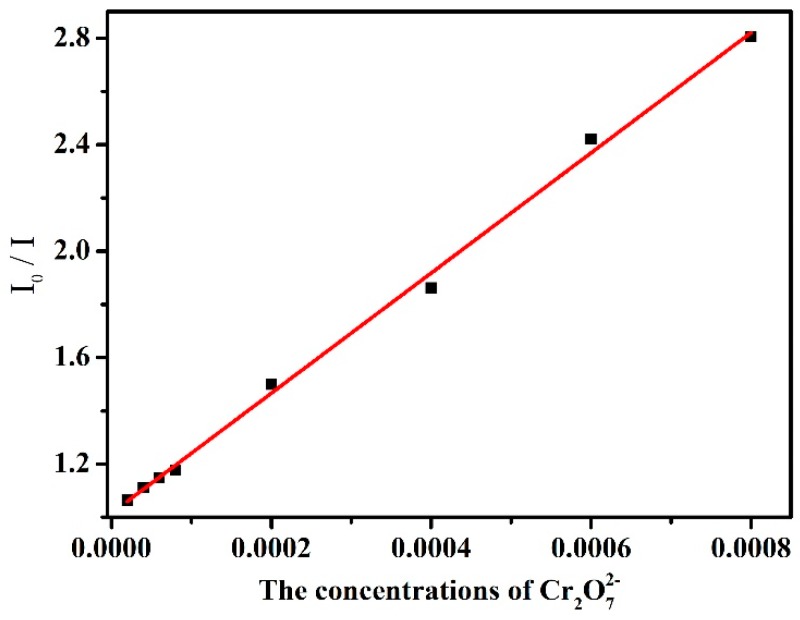
Stern–Volmer plot of compound **2** with different concentrations of Cr_2_O_7_^2−^.

Furthermore, we used the Stern–Volmer (S–V) equation to generate a plot of fluorescence intensity vs the concentration of Cr_2_O_7_^2−^ in the low concentration range of 0–10^–3^ mol/L, *I*_0_/*I* = *K*_SV_ [Cr_2_O_7_^2−^] + 1 (*K*_SV_ is quenching rate constant, and [Cr_2_O_7_^2−^] represents the concentration of Cr_2_O_7_^2−^). The S–V plot reveals that there exists a nearly linear correlation at low concentrations (*R*^2^ = 0.9972), which could be fitted as *I*_0_/*I* = *K*_SV_ [Cr_2_O_7_^2−^] + 1.01. The *K*_SV_ value is calculated to be 2.26 × 10^3^ L/mol (Figure 7), indicating that the Cr_2_O_7_^2−^ has a high quenching efficiency on the luminescence of compound **2**.

### 3.5. Catalytic Performance

Recently, it has been reported that Tb^3+^ sites in Tb-based MOFs could act as Lewis acidic centers [39]. Therefore, we explored the catalytic activity of compound **2**, and here the conversion of CO_2_ into cyclic carbonates was studied. The catalytic performance of compound **2** was investigated by using propylene oxide (PO) as the model substrate to explore the optimized catalytic reaction condition, and the corresponding results were shown in Table 1. The catalytic reaction was firstly conducted using compound **2** (2 mol%) and tetra-*n*-butyl-ammonium bromide (*n*-Bu_4_NBr, 2.5 mol%) as co-catalyst under relatively mild condition (0.2 MPa and 30 °C). The desired product propylene carbonate (PC) was obtained after different reaction time (12 h, 24 h, and 36 h), and the yield could reach 4.7%, 8.3%, and 9.9%, respectively (entries 1, 2, and 3 in Table 1). The results indicated that catalytic activity of compound **2** was poor under these conditions. Subsequently, the reaction pressure was increased to 1.0 MPa, the PO conversion reached 42.8% (entry 4). When the reaction temperature was increased to 70 °C and the pressure was kept at 1.0 MPa, the reaction could be completed within 12 h. In these preliminary studies, the results indicated that the higher temperature and pressure could facilitate the reaction process. 

**Figure 8:**
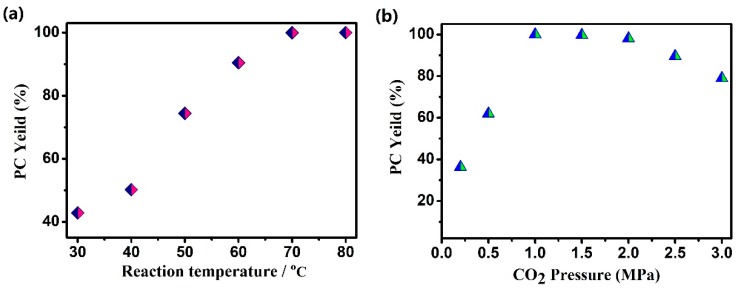
Influence of (**a**) reaction temperature (*P*(CO_2_) = 1.0 MPa, *t* = 12 h), and (**b**) CO_2_ pressure (*T* = 70 °C, *t* = 12 h) on the cyclo-addition of CO_2_ with PO. Other reaction conditions: PO (20 mmol); compound **2** (2 mol%); *n*-Bu_4_NBr (2.5 mol%).

As shown in Figure 8a, PO conversion increased from 30 to 70 °C under 1.0 MPa pressure with the reaction time of 12 h. Further increasing temperature, the PO conversion was not obviously increased. In addition, the yield increased with the prolonged reaction time. It is noteworthy that the reaction time was prolonged to 24 h, the reaction could be almost completed at a lower temperature of 60 °C (entry 5 in Table 1). CO_2_ pressure was also important to influence the cycloaddition of PO and CO_2_. As is seen from Figure 8b, the PO conversion increased with CO_2_ pressure 0.2 to 1 MPa and leveled in the pressure range of 1–2 MPa. Further increasing CO_2_ pressure (>2 MPa) could reduce the PC yield, which might be ascribed to the disruption of the lattice structure at a high pressure. In addition, the yields were very low when compound **2** or *n*-Bu_4_NBr was solely used as catalyst under the conditions of 1.0 MPa and 70 °C for 12 h (entries 7 and 8). However, the PO conversion was remarkably enhanced when compound **2** and *n*-Bu_4_NBr were employed simultaneously. It is known that compound **2** and *n*-Bu_4_NBr have excellent synergetic effect in the catalytic system of CO_2_ cyclo-addition reaction [40,41,42,43]. In conclusion, the optimized reaction conditions should be 70 °C and 1.0 MPa CO_2_ pressure with 2 mol% catalyst **2** and 2.5 mol% co-catalyst *n*-Bu_4_NBr for 12 h.

To further explore the catalytic performances of compound **2**, different functional group substituted epoxides were employed for CO_2_ cyclo-addition reaction under optimized reaction conditions. As showed in Table 2, the yields of corresponding cyclic carbonates decreased with the increasing of the alkyl length, which is probably due to the steric hindrance (entries 1–4). The cyclo-addition reactions of 1,2-epoxybutane and epichlorohydrin were catalyzed with high yield (entries 2 and 3), which also demonstrated effective catalytic performance of compound **2**. When styrene oxide was used as the reactant, the desired carbonate was generated with 88.4% yield (entry 4). However, the internal epoxide 1,2-epoxycyclohexane was converted to the corresponding cyclic carbonate with low yield (43.2%), which may be attributed to the high steric hindrance of cyclohexene oxide [44]. As a heterogeneous catalyst, recyclability is an essential feature to be considered in industrial applications. The recycling experiments were performed by using propylene oxide as substrate, and the results revealed that there was only 5.8% decrease of the catalytic activity after four catalytic cycles (Table 1, entry 9; Figures S6 and S7). The recycled Tb-MOF was characterized by PXRD investigation, and the result showed the framework of compound **2** could be well consistent with original one (Figure S5).

**Scheme 1:**
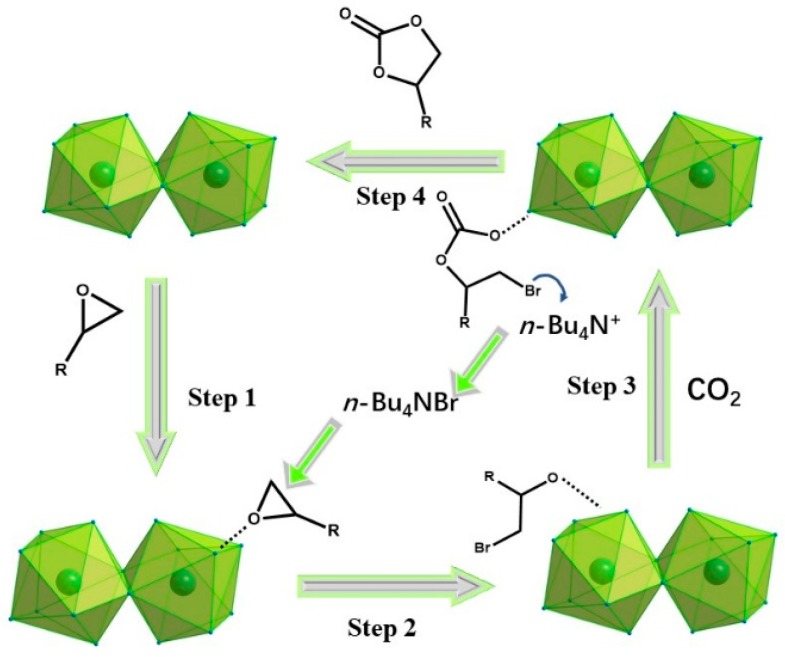
The proposed mechanism for cyclo-addition reaction of CO_2_ with epoxides using compound **2** and *n*-Bu_4_NBr as a co-catalyst.

A possible catalytic mechanism for Tb-MOF/*n*-Bu_4_NBr-catalyzed cyclo-addition of epoxides and CO_2_ was discussed based on reported studies (Scheme 1) [45,46]. The framework of compound **2** could enrich CO_2_ and epoxides owing to its porous channels, which might be beneficial for the reaction. The Lewis acidic site (Tb ions) interacted with the oxygen atom of the epoxide, activating the epoxy ring (Step 1). Then, the Br^−^ nucleophile from *n*-Bu_4_NBr attacks the less hindered carbon atom of the activated epoxide, promoting the ring opening of the epoxide to form active oxygen anion (Step 2). Subsequently, this active oxygen anion reacted with CO_2_ to produce alkylcarbonate salt (Step 3). Finally, the alkylcarbonate anion was converted to corresponding cyclic carbonate by ring closure and the co-catalyst *n*-Bu_4_NBr was regenerated simultaneously (Step 4).

## 4. Conclusions

In summary, two novel lanthanide metal–organic frameworks based on semi-rigid tripodal ligand 5-(4-carboxybenzyloxy) isophthalic acid (H_3_L) have been solvothermally synthesized and characterized. Compounds **1** and **2** are isostructural and possess 3D frameworks with ***flu***-3,6-*C*2/*c* structure topology, built from 6-connecting dinuclear metal–carboxylate unit and 3-connecting L^3^^−^ ligand. Photoluminescence measurement reveals that compound **1** shows near-infrared (NIR) emission, and compound **2** displays a selective fluorescence quenching response to Cr(VI) anion in liquid suspension. Owing to the accessible Lewis acidic sites in channels, compound **2** could be an efficient heterogeneous catalyst for cyclization reaction with epoxides and CO_2_, and the recycling number could reach four times without compromising catalytic activity. 

## Figures and Tables

**Table 1:**
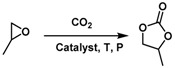
Cyclo-addition of CO_2_ with propylene oxide under different reaction conditions.

Entry	Temperature (°C)	Pressure (MPa)	Time (h)	Yield (%)
1	30	0.2	12	4.7
2	30	0.2	24	8.3
3	30	0.2	36	9.9
4	30	1.0	12	42.8
5	60	1.0	24	98.7
6	70	1.0	12	>99
7 ^[a]^	70	1.0	12	10.7
8 ^[b]^	70	1.0	12	17.3
9 ^[c]^	70	1.0	12	94.2

Reaction conditions: PO (20 mmol), compound 2 (2 mol%), *n*-Bu_4_NBr (0.5mmol, 2.5 mol%) under different conditions. Determined by ^1^H NMR spectroscopy. ^[a]^ In the absence of *n*-Bu_4_NBr. ^[b]^ In the absence of **2**. ^[c]^ The yield of the 4^th^ cycling.

**Table 2:**
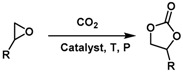
Cyclo-addition of CO_2_ with various substrates ^[a]^.

Entry	Substrate	Products	Yield (%) ^[b]^
1	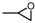	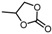	>99.0
2	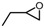	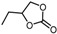	>99.0
3	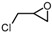	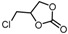	90.8
4	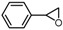	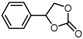	88.4
5	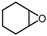	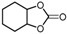	43.2

^[a]^ Reaction conditions: epoxides (20 mmol), compound **2** (2 mol%), *n*-Bu_4_NBr (2.5 mol%), CO_2_ (1 MPa), 70 °C, 12 h; and ^[b]^ determined by ^1^H NMR spectroscopy.

## Supplementary Materials

The following are available online at https://www.mdpi.com/2073-4360/11/5/868/s1, Materials and Instruments, X-Ray crystallography, Catalytic reactions. Figure S1: TGA profiles of compounds **1** and **2**; Figure S2: The emission spectra of ligand H_3_L; Figure S3: Solid state luminescence spectra of compound **2**; Figure S4: UV-Vis absorption spectra of compounds **1** and **2** aqueous solutions; Figure S5: The powder XRD patterns of compound **1**; Figure S6: The powder XRD patterns of compound **2**; Figure S7: Reusability of compound **2** on the PO conversion; Table S1: Crystallographic data and structure refinement details; Table S2: Selected bond lengths (Å) and angles (deg), ^1^H NMR characterization data.
