# Supplementary material for: Two Lanthanide Metal–Organic Frameworks Based on Semi-Rigid T-Shaped Tricarboxylate Ligand: Syntheses, Structures, and Properties
Source: Polymers (Basel). 2019 May 13;11(5):868. doi: 10.3390/polym11050868 (PMC6572171; doi:10.3390/polym11050868)
Supplement: Supplementary file 1 [file polymers-11-00868-s001.pdf]

Supporting Information

Two Lanthanide Metal–Organic Frameworks Based on Semi-rigid T-shaped Tricarboxylate Ligand: Syntheses, structures and properties

Yun-Shan Xue,[1,*] Zhuo-Lin Chen,[1] Youzhen Dong,[1] Wei-Wei Cheng[2]

1 School of Chemistry and Environmental Engineering, Yancheng Teachers University, Yancheng, Jiangsu 224051, P. R. China

2 State Key Laboratory of Materials-oriented Chemical Engineering, Nanjing Tech University, Nanjing 210009, P. R. China

3 School of Chemistry and Bioengineering, Nanjing Normal University Taizhou College, Taizhou 225300, P. R. China

Table of Contents

1. **Materials and Instruments**
2. **X-Ray crystallography**
3. **Catalytic reactions**
4. **Figures**

   Figure S1. TGA profiles of compounds **1-2**.

   Figure S2. The emission spectra of ligand H_3L.

   Figure S3. Solid state luminescence spectra of compound **2**.

   Figure S4. UV-Vis absorption spectra of compounds **1-2** aqueous solutions.

   Figure S5. The powder XRD patterns of compound **1**: (a) calculated, (b) as-synthesized.

   Figure S6. The powder XRD patterns of compound **2**: (a) calculated, (b) as-synthesized, (c) after 4th catalytic run.

   Figure S7. Reusability of compound **2** on the PO conversion.
5. **Tables**

   Table S1. Crystallographic data and structure refinement details for compounds **1** and **2**.

   Table S2. Selected bond lengths (Å) and angles (deg) for compounds **1** and **2**.
6. **^{1} H NMR characterization data**
7. **Reference**

Materials and Instruments.

All the chemicals were commercially purchased and used without further purification. Fourier transformed Infrared (FT-IR) spectra were obtained on a Bruker Vector 22 spectrophotometer with KBr pellets in the 4000–400 cm^{-1} region. Thermogravimetric analyses (TGA) were performed on a Perkin-Elmer thermal analyzer under nitrogen at a heating rate of 10 °C/min. Elemental analyses for C, H, N were obtained on an Elementar Vario MICRO Elemental Analyzer. Powder X-ray diffraction (PXRD) patterns were collected with a scan speed of 0.1 deg/s on a Bruker D8 Advance instrument using a Cu Kα radiation (λ = 1.54056 Å) at room temperature. Photoluminescence measurements were performed on a Perkin Elemer LS 55 Fluorescence Spectrophotometer. ^{1}H NMR spectra were carried out in $CDCl_3$ solvent on a Bruker 400 MHz spectrometer.

X-Ray crystallography.

X-ray single-crystal diffraction data were collected on a Bruker Apex II CCD with Mo-Kα radiation (λ = 0.7173 Å) using ω–2θ scan method. The crystal structures were solved by direct method and refined by full-matrix least-square techniques on F^2 using the SHELXTL crystallographic software package [1]. All of the non-hydrogen atoms were refined anisotropically. The hydrogen atoms of organic ligands were placed in geometrically calculated positions and refined using the riding model. The crystallographic data and structure refinement parameters of compounds **1** and **2** are given in Table S1, while selected interatomic bond lengths and angles with their estimated standard deviations are given in Table S2.

Catalytic reactions.

The catalytic reactions were carried out in 25 mL stainless steel high-pressure reactor. The activated catalyst (63.5 mg, about 2 mol %, based on Tb_2 clusters) together with the epoxide (20 mmol) and co-catalyst of tetra-*n*-tertbutylammonium bromide (*n*-Bu_4NBr, 161.1 mg, 0.5 mmol, 2.5 mol %) were transferred to the reactor immediately. The reactor was pressurized with CO_2 up to 1.0 MPa and stirred at 70 °C for 12 h. When the reaction was completed, the reactor was quickly cooled in ice water. For the catalyst recycling test, the catalyst was isolated by filtration and washed several times with EtOH and CH_2Cl_2 to fully remove the substrates, then dried under vacuum and reused in another catalytic experiment. The yield of product was determined by ^{1}H NMR spectroscopy and calculated from ^{1}H NMR according to the following equation [2].

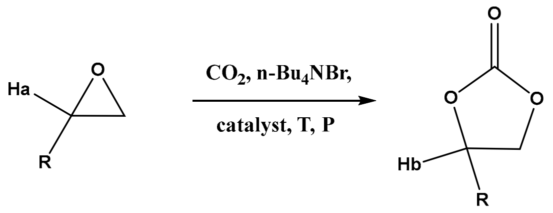

$$\text{yield} = \frac{{}^{1}H_b}{{}^{1}H_a + {}^{1}H_b}$$

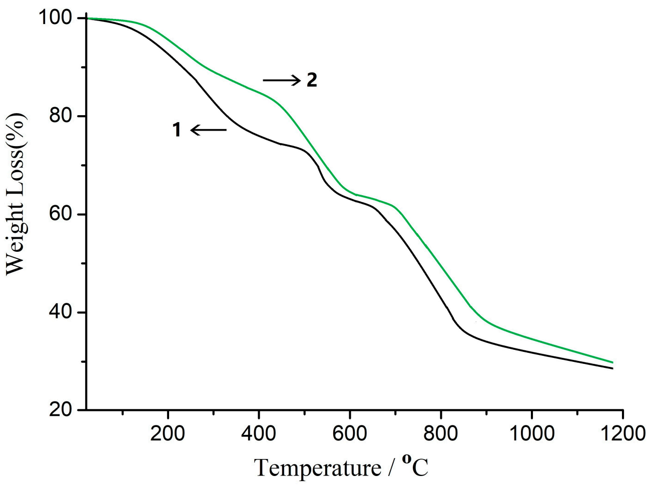

Figure S1. TGA profiles of compounds **1** and **2**.

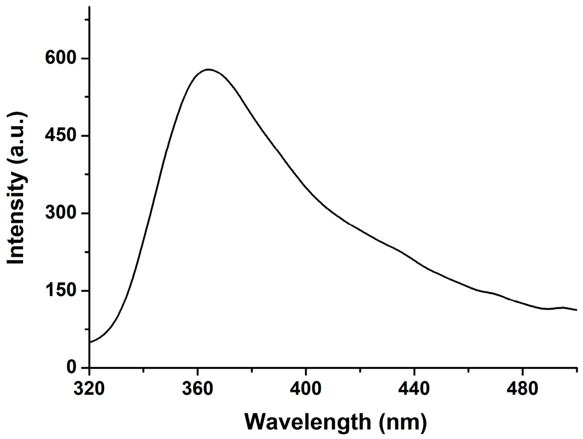

Figure S2. The emission spectra of ligand H_3L.

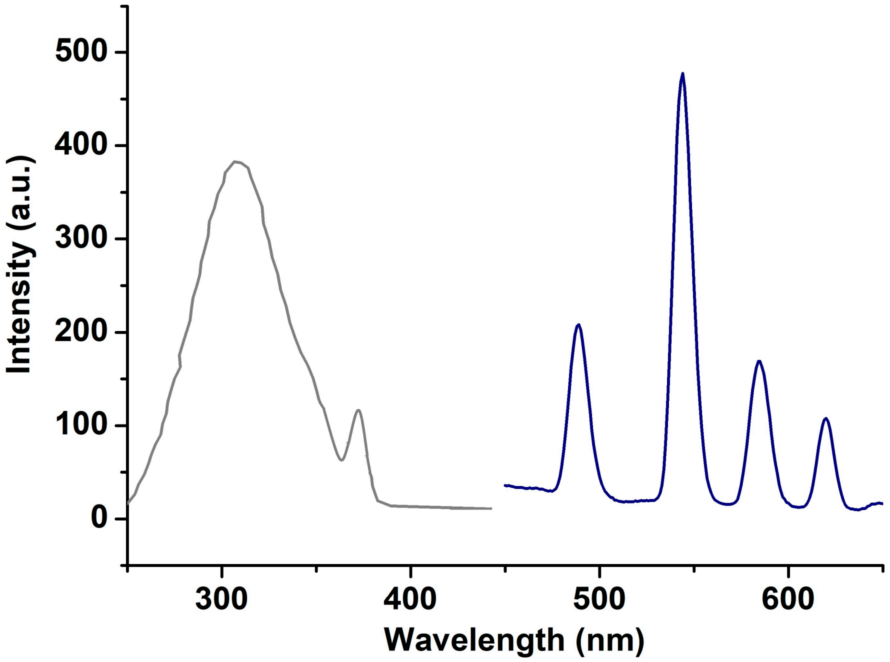

Figure S3. Solid state luminescence spectra of compound **2**.

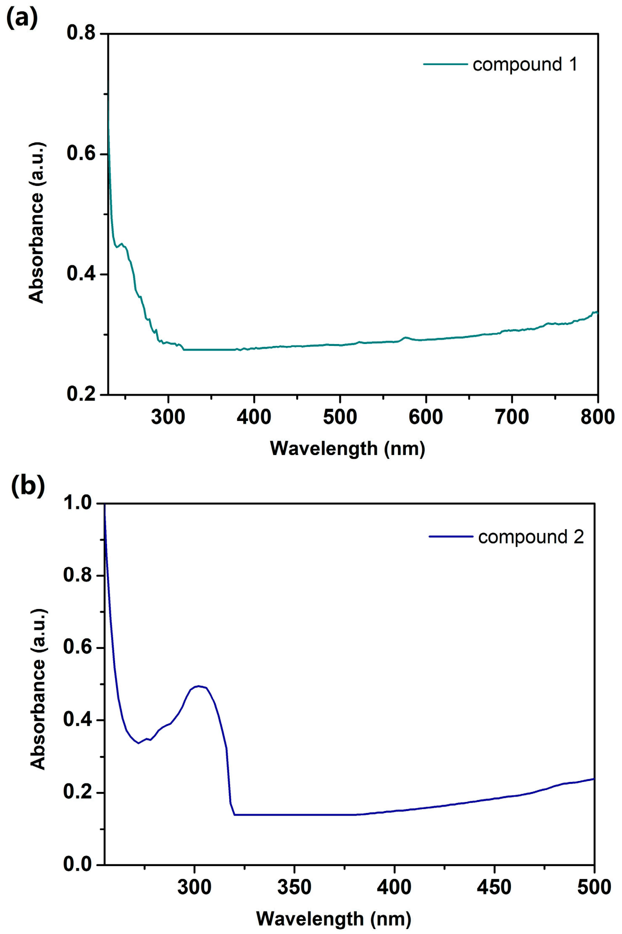

Figure S4. UV-Vis absorption spectra of compounds **1** and **2** aqueous solutions.

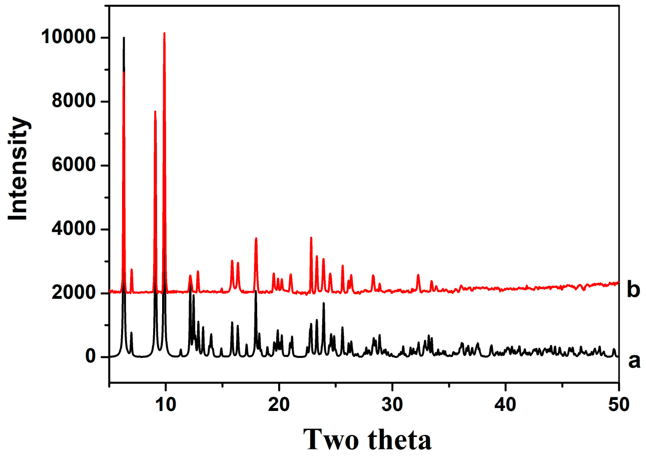

Figure S5. The powder XRD patterns of compound **1**: (**a**) calculated and (**b**) as-synthesized.

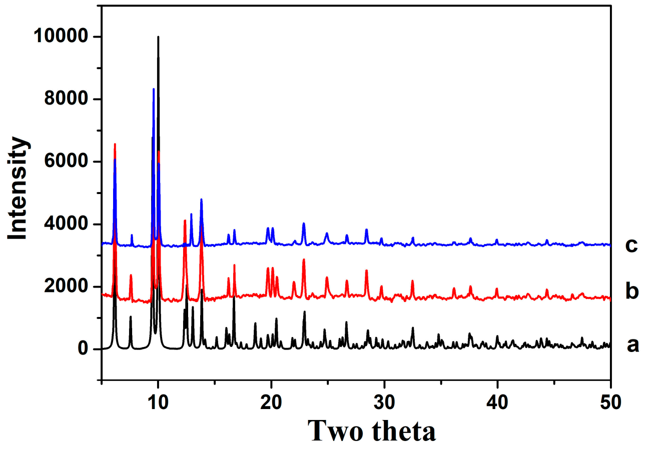

Figure S6. The powder XRD patterns of compound **2**: (**a**) calculated, (**b**) as-synthesized, and (**c**) after 4th catalytic run.

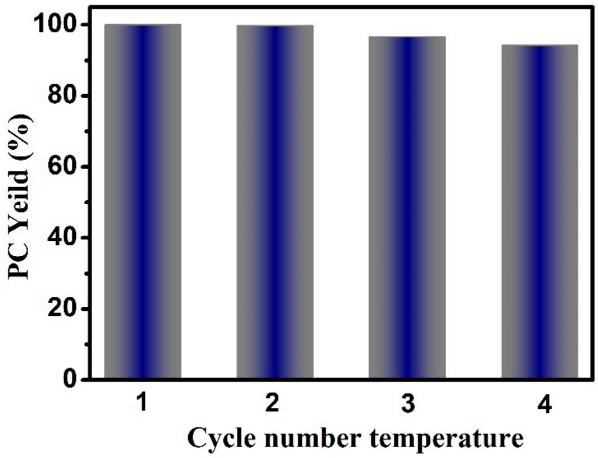

Figure S7. Reusability of compound **2** on the PO conversion.

Table S1. Crystal data and structure refinements for compounds **1** and **2**.

Compound reference	**1**	**2**
Chemical formula	$C_{47}H_{50}N_5Nd_2O_{19}$	$C_{19}H_{18}NO_9Tb$
Formula Mass	1277.40	563.26
Crystal system	Monoclinic	Monoclinic
a / Å	28.5153(16)	28.919(3)
b / Å	12.7727(14)	12.7727(14)
c / Å	13.9594(8)	14.4556(16)
α / °	90	90
β / °	100.292(2)	97.537(4)
γ / °	90	90
Unit cell volume / Å^3	5566.9(6)	5293.3(10)
Temperature / K	150.03	293(2)
Space group	*C*2/*c*	*C*2/*c*
Z	4	8
No. of reflections measured	46362	40839
No. of independent reflections	5708	6114
R_{int}	0.0642	0.0429
F(000)	2556	2208
Limits of data collection /°	2.272 □ 26.445	3.007 □ 27.578
μ(mm^{-1})	1.918	2.712
Final R_1 values ($I > 2\sigma(I)$)	0.0460	0.0225
Final $wR(F^2)$ values ($I > 2\sigma(I)$)	0.1225	0.0567
Final R_1 values (all data) [a]	0.0585	0.0306
Final $wR(F^2)$ values (all data) [b]	0.1290	0.0595
Goodness of fit on F^2	1.053	1.043
CCDC	1907632	1907633

[a] $R_1 = \Sigma||F_o| - |F_c||/\Sigma|F_o|$, [b] $wR = [\Sigma w(F_o^2 - F_c^2)^2/\Sigma w(F_o^2)^2]^{1/2}$

Table S2. Selected bond distances (Å) and angles (°) for compounds **1** and **2**.

compound **1**			
Nd(1)-O(1)#3	2.627(4)	Nd(1)-O(5)#2	2.674(4)
Nd(1)-O(2)#3	2.444(4)	Nd(1)-O(5)#4	2.419(4)
Nd(1)-O(4)#2	2.517(4)	Nd(1)-O(6)#1	2.450(4)
Nd(1)-O(7)	2.426(4)	Nd(1)-O(8)	2.428(5)
Nd(1)-O(9)	2.500(5)		
O(1)#3-Nd(1)-Nd(1)#1	117.24(11)	O(1)#3-Nd(1)-O(5)#2	139.89(14)
O(2)#3-Nd(1)-Nd(1)#1	147.49(13)	O(2)#3-Nd(1)-O(4)#2	82.18(15)
O(2)#3-Nd(1)-O(6)#1	140.80(16)	O(2)#3-Nd(1)-O(5)#2	128.91(15)
O(2)#3-Nd(1)-O(1)#3	51.42(14)	O(2)#3-Nd(1)-O(9)	71.65(17)
O(4)#2-Nd(1)-Nd(1)#1	84.50(9)	O(4)#2-Nd(1)-O(5)#2	50.12(12)
O(4)#2-Nd(1)-O(1)#3	124.54(14)	O(5)#2-Nd(1)-Nd(1)#1	35.52(8)
O(5)#4-Nd(1)-Nd(1)#1	39.95(9)	O(5)#4-Nd(1)-O(7)	73.35(14)
O(5)#4-Nd(1)-O(4)#2	123.28(14)	O(5)#4-Nd(1)-O(2)#3	135.31(14)
O(5)#4-Nd(1)-O(6)#1	79.30(14)	O(5)#4-Nd(1)-O(5)#2	75.47(14)
O(5)#4-Nd(1)-O(8)	82.35(18)	O(5)#4-Nd(1)-O(1)#3	85.26(13)
O(5)#4-Nd(1)-O(9)	145.28(16)	O(6)#1-Nd(1)-Nd(1)#1	69.12(10)
O(6)#1-Nd(1)-O(4)#2	92.49(16)	O(6)#1-Nd(1)-O(5)#2	68.33(14)
O(6)#1-Nd(1)-O(1)#3	142.18(15)	O(6)#1-Nd(1)-O(9)	69.54(16)
O(7)-Nd(1)-Nd(1)#1	66.92(9)	O(7)-Nd(1)-O(4)#2	74.49(15)
O(7)-Nd(1)-O(2)#3	80.95(16)	O(7)-Nd(1)-O(6)#1	135.03(14)
O(7)-Nd(1)-O(5)#2	70.63(13)	O(7)-Nd(1)-O(8)	134.00(16)
O(7)-Nd(1)-O(1)#3	70.24(15)	O(7)-Nd(1)-O(9)	140.63(17)
O(8)-Nd(1)-Nd(1)#1	115.08(15)	O(8)-Nd(1)-O(4)#2	149.18(17)
O(8)-Nd(1)-O(2)#3	90.5(2)	O(8)-Nd(1)-O(6)#1	74.50(17)
O(8)-Nd(1)-O(5)#2	139.43(16)	O(8)-Nd(1)-O(1)#3	69.32(16)
O(8)-Nd(1)-O(9)	75.1(2)		
compound **2**			
Tb(1)-O(1)#3	2.542(2)	Tb(1)-O(2)#3	2.3950(19)
Tb(1)-O(4)	2.3798(18)	Tb(1)-O(5)#1	2.3607(18)
Tb(1)-O(6)#4	2.3277(18)	Tb(1)-O(6)#2	2.6694(18)
Tb(1)-O(7)#2	2.4314(19)	Tb(1)-O(8)	2.430(2)
Tb(1)-O(9)	2.381(2)		
O(1)#3-Tb(1)-Tb(1)#1	116.46(6)	O(1)#3-Tb(1)-O(6)#2	138.95(7)
O(2)#3-Tb(1)-O(7)#2	79.84(7)	O(2)#3-Tb(1)-O(6)#2	126.45(7)
O(2)#3-Tb(1)-O(8)	71.62(8)	O(2)#3-Tb(1)-O(1)#3	52.48(7)
O(2)#3-Tb(1)-Tb(1)#1	143.47(5)	O(4)-Tb(1)-Tb(1)#1	67.35(4)
O(4)-Tb(1)-O(7)#2	71.82(7)	O(4)-Tb(1)-O(2)#3	76.60(7)
O(4)-Tb(1)-O(6)#2	70.30(6)	O(4)-Tb(1)-O(8)	134.81(8)
O(4)-Tb(1)-O(1)#3	70.31(7)	O(4)-Tb(1)-O(9)	136.63(9)
O(5)#1-Tb(1)-Tb(1)#1	68.70(5)	O(5)#1-Tb(1)-O(7)#2	95.02(8)
O(5)#1-Tb(1)-O(2)#3	144.74(7)	O(5)#1-Tb(1)-O(6)#2	68.69(6)

O(5)#1-Tb(1)-O(4)	135.18(6)	O(5)#1-Tb(1)-O(8)	73.66(8)
O(5)#1-Tb(1)-O(1)#3	140.53(8)	O(5)#1-Tb(1)-O(9)	74.99(9)
O(6)#4-Tb(1)-Tb(1)#1	40.27(4)	O(6)#2-Tb(1)-Tb(1)#1	34.30(4)
O(6)#4-Tb(1)-O(5)#1	78.20(7)	O(6)#4-Tb(1)-O(7)#2	122.27(6)
O(6)#4-Tb(1)-O(2)#3	133.87(7)	O(6)#4-Tb(1)-O(6)#2	74.57(6)
O(6)#4-Tb(1)-O(4)	74.21(6)	O(6)#4-Tb(1)-O(8)	149.78(7)
O(6)#4-Tb(1)-O(1)#3	84.11(7)	O(6)#4-Tb(1)-O(9)	87.09(9)
O(7)#2-Tb(1)-Tb(1)#1	83.49(4)	O(7)#2-Tb(1)-O(6)#2	50.60(6)
O(7)#2-Tb(1)-O(1)#3	124.10(7)	O(8)-Tb(1)-Tb(1)#1	132.33(7)
O(8)-Tb(1)-O(7)#2	71.62(9)	O(8)-Tb(1)-O(6)#2	104.53(8)
O(8)-Tb(1)-O(1)#3	111.16(9)	O(9)-Tb(1)-Tb(1)#1	119.83(7)
O(9)-Tb(1)-O(7)#2	146.91(9)	O(9)-Tb(1)-O(2)#3	90.37(9)
O(9)-Tb(1)-O(6)#2	141.82(8)	O(9)-Tb(1)-O(8)	75.30(10)
O(9)-Tb(1)-O(1)#3	69.08(8)		

Symmetry transformations: for **1** #1 -x+0.5, -y+1.5, -z+1, #2 -x+0.5, y+0.5, -z+0.5, #3 -x+1, y, -z+0.5, #4 x, -y+1, z+0.5, #5 -x+0.5, y-0.5, -z+0.5, #6 x, -y+1, z-0.5, #7 -x+0.5, -y+2.5, -z+1; for **2** #1 -x+1.5, -y+0.5, -z+1, #2 -x+1.5, y+0.5,-z+1.5, #3 -x+1, y, -z+1.5, #4 x, -y, z-0.5, #5 -x+1.5, y-0.5, -z+1.5, #6 x, -y, z+0.5

^{1}H NMR characterization data:

4-Methyl-1.3-dioxolan-2-one:

^{1}H NMR ($CDCl_3$, 400 MHz) δ 1.48 (d, 3H), 3.99-4.03 (m, 1H), 4.53-4.57 (m, 1H), 4.81–4.90 (m, 1H).

4-Ethyl-1,3-dioxolan-2-one:

^{1}H NMR ($CDCl_3$, 400 MHz) δ 1.02 (t, 3H), 1.80 (m, 2H), 4.08 (dd, 1H), 4.52 (t, 1H), 4.62–4.69 (m, 1H).

4-Chloromethyl-1,3-dioxolan-2-one:

^{1}H NMR ($CDCl_3$, 400 MHz) δ 3.70-3.80 (m, 2H), 4.42 (q, 1H), 4.59 (t, 1H), 4.93–4.99 (m, 1H).

4-Phenyl-1,3-dioxolan-2-one:

^{1}H NMR ($CDCl_3$, 400 MHz) δ 4.33 (t, 1H), 4.82 (t, 1H), 5.69 (t, 1H), 7.26–7.29 (m, 2H), 7.31-7.37 (m, 3H).

Hexahydrobenzo[d][1,3]dioxol-2-one:

^{1}H NMR ($CDCl_3$, 400 MHz) δ 1.37–1.49 (m, 4H), 1.87–1.97 (m, 4H), 5.29 (m, 2H).

Reference

1 Sheldrick G.M. *Acta Crystallogr. Sect. C*, **2015**, *C71*, 3-5.

2 Ai, J.; Min, X.; Gao, C.Y.; Tian, H.R.; Dang, S.; Sun, Z.M. *Dalton Trans*. **2017**, *46*, 6756-6761.
